# Epidemiology, Pathogenesis, and Clinical Approach in Group 5 Pulmonary Hypertension

**DOI:** 10.3389/fmed.2020.616720

**Published:** 2021-03-25

**Authors:** Mazen Al-Qadi, Barbara LeVarge, H. James Ford

**Affiliations:** Division of Pulmonary and Critical Care Medicine, Pulmonary Hypertension Program, University of North Carolina at Chapel Hill, Chapel Hill, NC, United States

**Keywords:** multifactorial, pulmonary hypertension, Group 5, hematologic, sickle cell, sarcoidosis, metabolic, CKD - chronic kidney disease

## Abstract

Pulmonary hypertension (PH) is recognized to be associated with a number of comorbid conditions. Based on these associations, PH is classified into 5 groups, considering common pathophysiologic drivers of disease, histopathologic features, clinical manifestations and course, and response to PH therapy. However, in some of these associated conditions, these characteristics are less well-understood. These include, among others, conditions commonly encountered in clinical practice such as sarcoidosis, sickle cell disease, myeloproliferative disorders, and chronic kidney disease/end stage renal disease. PH in these contexts presents a significant challenge to clinicians with respect to disease management. The most recent updated clinical classification schemata from the 6th World Symposium on PH classifies such entities in Group 5, highlighting the often unclear and/or multifactorial nature of PH. An in-depth review of the state of the science of Group 5 PH with respect to epidemiology, pathogenesis, and management is provided. Where applicable, future directions with respect to research needed to enhance understanding of the clinical course of these entities is also discussed.

## Introduction

Pulmonary hypertension (PH) is characterized by elevated mean pulmonary artery pressure (mPAP) of >20 mmHg as determined by right heart catheterization (RHC). PH is divided into 5 groups based on the underlying mechanism using the original World Health Organization classification system framework. In 2019, the proceedings of the World Symposium on Pulmonary Hypertension published the updated and revised classification based on the 6th World Symposium on PH (1). According to the new classification, causes of PH include pulmonary arterial hypertension (PAH, Group 1 PH), pulmonary venous hypertension due to elevated filling pressure of the left heart (Group 2 PH), PH due to chronic pulmonary disease or hypoxemia (Group 3 PH), chronic thromboembolic disease (Group 4), whereas PH that develops due to multiple or unclear mechanisms is referred to as Group 5 PH (Table 1). Within Group 5, several clinical disorders are implicated in association with the development of PH (Table 2). The most commonly seen clinical entities in Group 5 PH (Groups 5.1–5.3) are covered in detail: PH in the setting of hematologic disorders, sarcoidosis, and chronic renal failure. A brief overview of some of the remaining, less common but clinically relevant entities in Group 5 is also provided. Complex congenital heart disease (Group 5.4) is outside the scope of this manuscript.

**Table 1:** Current clinical classification of pulmonary hypertension (1).

**Group**	**Description**
1	**Pulmonary Arterial Hypertension** 1.1 Idiopathic PAH 1.2 Heritable PAH 1.3 Drug- and toxin-induced PAH 1.4 PAH associated with:
	1.4.1 Connective tissue disease 1.4.2 HIV infection 1.4.3 Portal hypertension 1.4.4 Congenital heart disease 1.4.5 Schistosomiasis
	1.5 PAH long-term responders to CCB 1.6 PAH with overt signs of venous/capillaries !!break (PVOD/PCH) involvement 1.7 Persistent PH of the Newborn syndrome
2	**PH Due to Left Heart Disease** 2.1 PH due to heart failure with preserved EF 2.2 PH due to heart failure with reduced EF 2.3 Valvular heart disease 2.4 Congenital/acquired CV conditions leading to post-capillary PH
3	**PH Due to Lung Diseases and/or Hypoxia** 3.1 Obstructive lung disease 3.2 Restrictive lung disease 3.3 Other lung disease with mixed restrictive/obstructive pattern 3.4 Hypoxia without lung disease 3.5 Developmental lung disorders
4	**PH Due to Pulmonary Artery Obstruction** 4.1 Chronic thromboembolic PH 4.2 Other pulmonary artery obstructions
5	**PH With Unclear and/or Multifactorial Mechanisms** 5.1 Hematologic disorders 5.2 Systemic disorders 5.3 Others 5.4 Complex congenital heart disease

**Table 2:** Subclassifications of group 5 pulmonary hypertension: PH with unclear and/or multifactorial mechanisms (1).

5.1 Hematologic disorders	Hemolytic anemias Myeloproliferative disorders
5.2 Systemic and metabolic disorders	Pulmonary Langerhans cell histiocytosis Gaucher disease Glycogen storage disease Neurofibromatosis Sarcoidosis
5.3. Others	Chronic renal failure with or without hemodialysis Fibrosing mediastinitis
5.4 Complex congenital heart diseases	Segmental pulmonary hypertension • Isolated pulmonary artery of ductal origin • Absent pulmonary artery • Pulmonary atresia with ventricular septal defect and major aorto-pulmonary collateral arteries • Hemitruncus • Other Single ventricle • Unoperated • Operated Scimitar syndrome

Considering the true upper limits of normal mPAP, the 6th World Symposium on PH introduced revised hemodynamic definitions, affirming PH in those with mPAP >20 mmHg as opposed to mPAP ≥25 mmHg as previously defined. A pulmonary vascular resistance (PVR) of at least 3 Wood Units was further mandatory to define pre-capillary pulmonary hypertension. These changes impact identification and management of all groups of PH, including group 5 PH. With updated review and discussion of the literature, the proceedings outlined additional changes specifically impacting group 5 PH, including removal of both splenectomy and thyroid disease as specific subtypes of PH (asserting these conditions as risk factors) and reclassification of lymphangioleiomyomatosis into Group 3 PH (1).

## Pulmonary Hypertension in Hematologic Disorders

### Chronic Hemolytic Anemias

#### Prevalence

PH is increasingly recognized as a major source of morbidity and mortality in patients with chronic hemolysis, most notably in the context of sickle cell disease. The prevalence of PH in hemolytic anemia is variable depending upon the diagnostic criteria, methods used to diagnose PH, and population studied. In sickle cell disease (SCD, hemoglobin SS), 30–40% of patients have evidence of elevated pulmonary artery pressures based on tricuspid regurgitant jet velocity (TRV) ≥2.5 m/s on Doppler echocardiography. However, only 6–10.5% of patients have mPAP of ≥25 mmHg on invasive hemodynamic evaluation by RHC (2–5). This reflects the fact that estimation of pulmonary artery pressure is often inaccurate by echocardiogram. Additionally, elevated pulmonary artery pressures can be related to high cardiac output (due to anemia) and not always secondary to increased pulmonary vascular resistance (PVR). Using the new definition of PH (mPAP >20 mmHg), the prevalence of PH in SCD is probably higher than that reported in these studies.

PH has also been reported in association with other hemolytic anemias. In a study of 110 patients with β-thalassemia intermedia, 59% of patients had elevated peak systolic tricuspid gradient >30 mmHg on Doppler echocardiography, suggestive of PH. Only 6 patients had RHC and were found to have severe pre-capillary PH. All patients in this study had normal left ventricular function and high cardiac output (6). The prevalence of PH by echocardiography was even higher (75%) in a small study of 35 patients with homozygous β-thalassemia (7). More recently, Derchi et al. evaluated the prevalence of PH in a large multicenter cross-sectional study of 1,309 patients with β-thalassemia in Italy. Nine percent of patients had TRV >3 m/s; patients in the “PH likely” group with TRV ≥3.2 m/s underwent RHC. The prevalence of RHC-confirmed PH was 2.1%. Increased age and splenectomy were independent risk factors for PH in this study (8).

Notably, the prevalence of PH in patients with hereditary spherocytosis is much lower than reported with SCD and thalassemia. In two retrospective studies, no patients had a TRV of ≥2.8 m/s on echocardiography to suggest PH (9, 10). Nonetheless, PH has been described in hereditary spherocytosis and stomatocytosis, with most cases related to chronic thromboembolic pulmonary hypertension (CTEPH) following splenectomy (11).

#### Pathogenesis

The pathogenesis of PH in chronic hemolysis is multifaceted and is most extensively studied in SCD. The vasomotor tone of the pulmonary circulation is normally regulated by nitric oxide (NO), a powerful vasodilator and modulator of endothelial proliferation. NO is produced by the pulmonary endothelium in response to shear stress on the surface of endothelial cells (12). The enzyme NO synthase catalyzes cleavage of the terminal amino group from the amino acid L-arginine, producing NO. Hemolysis causes the release of arginase-1, which depletes L-arginine eliminating the NO precursor (13). Additionally, studies have shown that free hemoglobin produced by intravascular hemolysis in SCD consumes NO (14, 15). Impaired NO production is also related to hemolysis-induced generation of asymmetric dimethylarginine, an endogenous NO synthase inhibitor (16). Production of heme due to oxidation of free hemoglobin also activates Toll-like receptor 4 promoting vaso-occlusive events (17, 18). Interestingly, a growing body of literature indicates a possible role for free hemoglobin in the pathogenesis of PAH in patients with no known hemolysis. Rafikova et al. found that free hemoglobin levels were significantly higher in patients with known PAH compared to patients with no PAH. Furthermore, the severity of PAH correlated with higher cell-free hemoglobin levels. In an animal model of PAH, inhibition of heme translocation using sulfasalazine prevented the development of PH (19).

Endothelin-1 (ET-1), a potent vasoconstrictor of pulmonary vascular bed, plays a role in the imbalance between vasodilators and vasoconstrictors in SCD. Plasma and urine levels of ET-1 are elevated in patients with SCD compared with matched controls (20). Furthermore, Phelan et al. showed that exposure of cultured human endothelial cells to previously sickled erythrocytes results in enhanced gene expression of ET-1 (21).

Hypercoagulability is another important factor that adds to pulmonary endothelial dysfunction. A hypercoagulable state results from reduced NO, asplenia (functional or surgical), and activation of thrombotic factors (tissue factor, platelets, and thrombin) in SCD (22, 23). This is supported by findings of pulmonary thromboemboli in 38–80% of postmortem lung examinations of deceased SCD patients (24, 25).

Proliferative arteriopathy seen in SCD-PH implies possible contribution of angiogenic factors to the pathogenesis. A recent case-control study of children with β-thalassemia major demonstrated a significantly higher serum level of vascular endothelial growth factor in patients with PH than those without PH or matched healthy children (26).

Finally, a significant number of patients with SCD have been found to have restrictive cardiomyopathy and diastolic dysfunction (27). In fact, 50% of patients with SCD and PH have pulmonary artery wedge pressure (PAWP) of >15 mmHg, consistent with pulmonary venous hypertension (i.e., post-capillary PH) (3, 5, 28). Vaso-occlusive events, systemic hypertension, iron overload (due to transfusions) and end-organ damage (e.g., renal failure) in patients with chronic hemolysis contribute to diastolic left ventricular dysfunction as well (29). Collectively, all these factors increase the risk of developing PH in patients with SCD and other hemolytic anemias.

#### Evaluation

**Figure 1:**
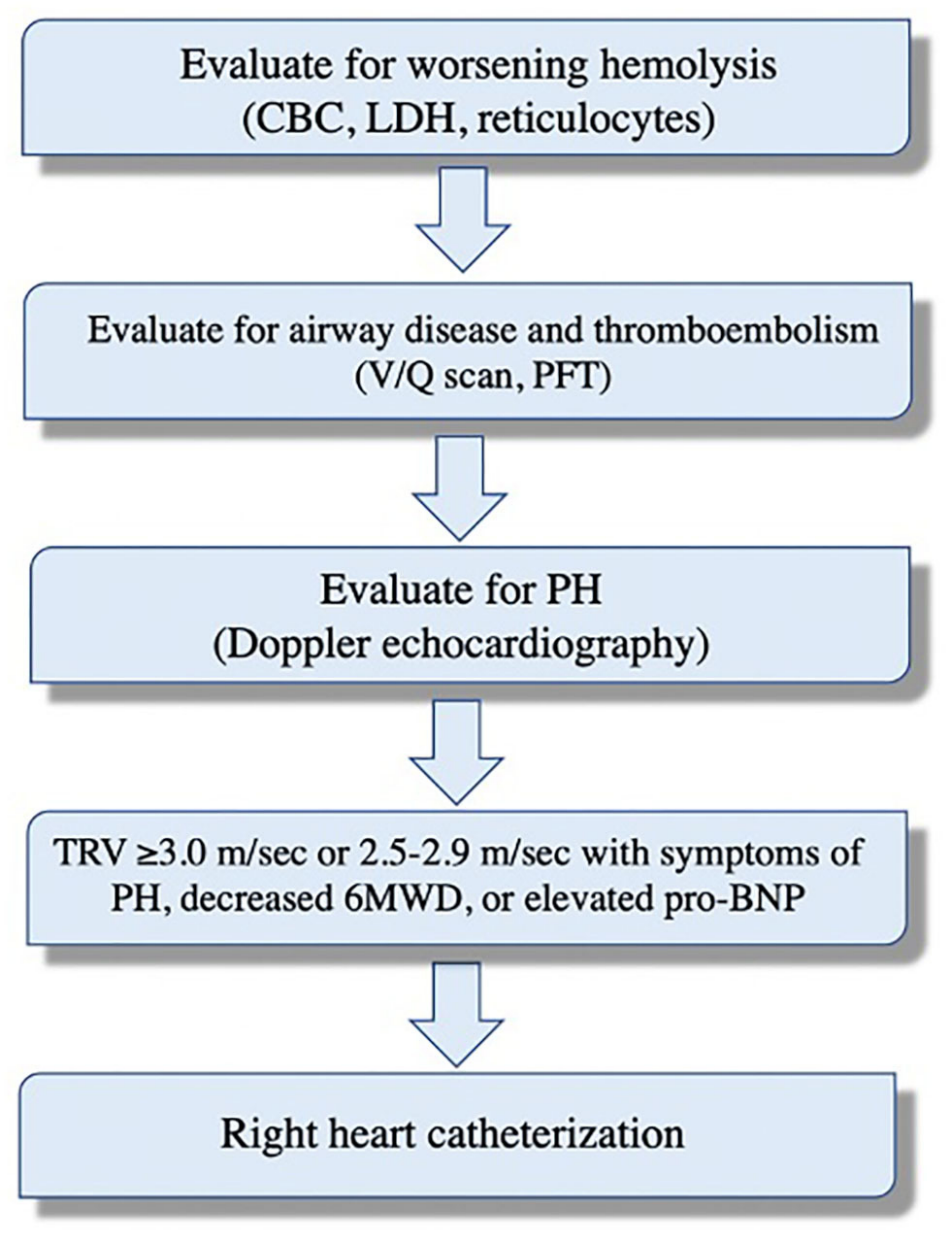
Systematic evaluation of dyspnea and potential pulmonary hypertension in patients with sickle cell disease.

Pulmonary symptoms are common in patients with chronic hemolysis and often are multifactorial. Patients with SCD, for example, may have recurrent chest pains, fatigue, and chronic shortness of breath. These symptoms can be related to anemia, acute chest syndrome, obstructive airway disease, thromboembolism, and/or PH (30–32). Therefore, these should be systematically evaluated (Figure 1). Asymptomatic patients also remain at greater risk of developing PH; therefore, screening with Doppler echocardiography is recommended. The American Thoracic Society guidelines recommend screening patients with SCD with Doppler echocardiography every 1–3 years to assess mortality risk. Importantly, echocardiography should not be performed within 4 weeks of acute chest syndrome or within 2 weeks of acute vaso-occlusive crisis as these acutely increase the pulmonary artery systolic pressure (PASP) due to hypoxemia and worsening anemia (33). There are no screening guidelines for patients with other hemolytic diseases. Nonetheless, it is reasonable to obtain echocardiography on high-risk patients (e.g., severe hemolysis, asplenia, prior venous thromboembolism, and iron overload), especially those with unexplained pulmonary symptoms (34).

**Figure 2:**
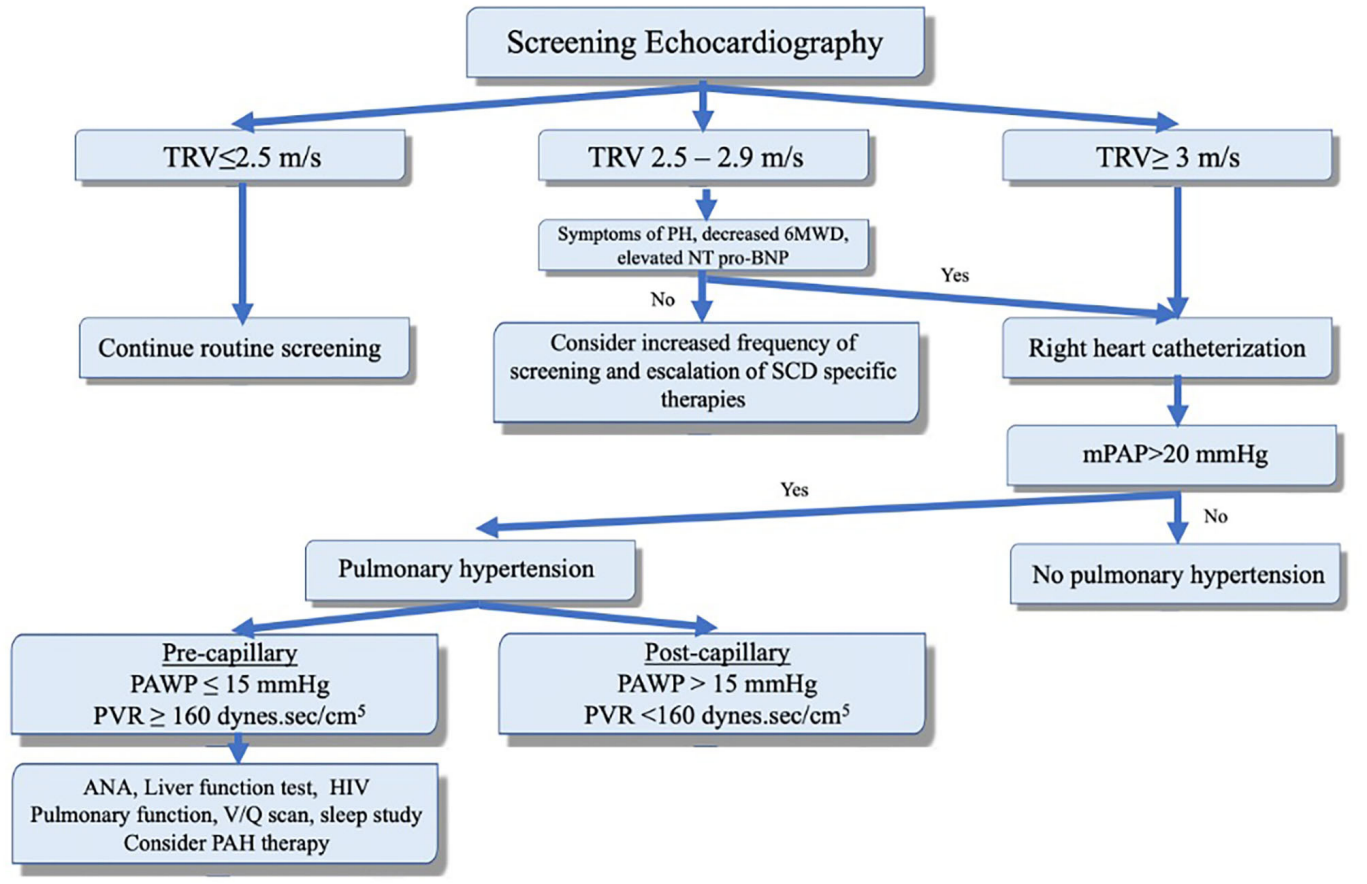
Screening, diagnosis, and clinical classification to guide management of pulmonary hypertension in the context of sickle cell disease. Adapted with permission of the American Thoracic Society. Copyright © 2020 American Thoracic Society. All rights reserved. Klings et al. (33). The American Journal of Respiratory and Critical Care Medicine is an official journal of the American Thoracic Society. Readers are encouraged to read the entire article for the correct context. The authors, editors, and The American Thoracic Society are not responsible for errors or omissions in adaptations.

PH is suspected if the TRV on echocardiography is >2.5 m/sec. However, TRV ≥2.5 m/s has a low positive predictive value as only 31% of such patients had mPAP ≥25 mmHg on RHC (27). A TRV ≥3 m/s is 3 standard deviations above the population mean and is more specific (PH confirmed in 66–77% of patients on RHC) (4, 5). A TRV between 2.5 and 3 m/s combined with either an NT-proBNP > 164.5 pg/ml or 6-min walk distance of < 333 m increases the likelihood of PH and warrants further evaluation and RHC (Figure 2) (3, 5). PASP is commonly estimated indirectly by measuring the right ventricular systolic pressure which (in the absence of pulmonic stenosis) are equivalent. Using the modified Bernoulli equation (*P* = 4V^2^), the PASP is calculated from the TRV. This means that minor variations in measurement of TRV can result in significant differences in estimated pulmonary pressures. Furthermore, even when accurate, elevated PASP can be related to high cardiac output often seen in patients with anemia. On the other hand, PH can be missed on echocardiography as the TRV is not always measurable (35). Thus, RHC remains the test of choice to confirm the presence of PH and assess the PAWP, cardiac output, and calculated PVR. It is worth mentioning that reduced blood viscosity and high cardiac output (due to anemia) result in a lower baseline PVR than healthy subjects. Thus, PVR of >2 Wood units is considered elevated in these patients (3–5).

Blood biomarkers can also be helpful in the evaluation of PH in chronic hemolytic anemia. Lactate dehydrogenase is a commonly used marker for hemolysis has been shown to correlate with NO resistance and severity of PH (36). NT-proBNP is another useful biomarker for PH and right ventricular dysfunction. In a study by Machado et al., elevated NT- proBNP (>160 pg/mL) in SCD patients correlated with TRV and was predictive of PH. Additionally, elevated NT- proBNP was an independent predictor of mortality (37).

#### Treatment

Management of PH in hemolytic anemias includes general measures such as supplemental oxygen to reverse hypoxemia and prevent its deleterious effect on the pulmonary vascular tone. Diuretics are used to treat volume overload but should be used cautiously due to risk of sickling in patients with SCD. Lifelong anticoagulation is indicated in SCD patients with PH secondary to CTEPH. Although surgical pulmonary thromboendarterectomy (PTE) can be curative in CTEPH, it is undoubtedly more challenging in SCD patients due to the increased risk of sickling and occlusive crises during cardiopulmonary bypass. The best perioperative approach in these patients remains controversial. Avoidance of hypoxemia, hypothermia, and acidosis reduces the risk of perioperative complications. Additionally, exchange transfusion to reduce hemoglobin S to <20% prior to surgery is advised (38, 39). This aggressive transfusion regimen, however, has not been shown to reduce perioperative complications in patients with SCD (40). Inoperable patients are considered for balloon pulmonary angioplasty (BPA). In a recent study of PH in SCD, three patients with CTEPH underwent BPA with significant reduction in PVR in 2 patients and normalization of mPAP in the third patient (41).

Optimizing treatment of the underlying hemolytic disease is of paramount importance. Hydroxyurea is a myelosuppressive agent that has been shown to decrease sickle cell hemoglobin polymerization, reducing hemolysis and frequency of acute chest syndrome, and vaso-occlusive crises. Furthermore, hydroxyurea decreases hospitalization and mortality in SCD patients with HbSS and is currently recommended for patients with more than one episode of acute chest syndrome or >3 vaso-occlusive crises per year (33, 42–44). The risk of developing PH is also reduced with hydroxyurea in patients with thalassemia (45). Chronic transfusion is recommended in SCD patients who fail to respond or cannot tolerate hydroxyurea. This strategy is associated with improvement in pulmonary vascular changes in SCD patients (46). Similarly, adherence to transfusion and iron chelation therapy in patients with thalassemia prevented PH (47, 48).

SCD patients who remain symptomatic despite optimization of their SCD therapy (i.e., hydroxyurea, transfusion) are considered for PH-targeted therapy. This is usually reserved for patients with moderate to severe (based on functional class) RHC-confirmed pre-capillary PH with relatively reduced cardiac output. In general, endothelin receptor antagonists (ERAs) are recommended in patients with moderate PH (Functional class 2-3). Bosentan, an endothelin receptor antagonist, was evaluated in two randomized placebo-controlled trials of SCD patients with RHC-proven pre- and post-capillary PH. Both studies were terminated early due to slow enrollment but modest improvement in hemodynamic parameters was observed. Efficacy endpoints were not analyzed to draw definitive conclusions but bosentan was well-tolerated (49). Patients with more severe PH (class IV functional status, evidence of right-sided heart failure, or reduced cardiac output) can be treated with prostanoids. The efficacy of prostacyclins has been evaluated in a small retrospective study of 11 patients who received treprostinil or epoprostenol and showed improvement in right ventricular systolic pressure on echocardiography (50). Patients who need such therapy should be referred to centers with expertise in managing PH in SCD patients due to concerns of complications (e.g., high-cardiac output pulmonary edema, line thrombosis). Phosphodiesterase type 5 inhibitors (PDE-5Is) should generally be avoided in this population due to risk of increased hospitalizations for painful crises noted in a study of sildenafil (51). Riociguat is a soluble guanylate cyclase stimulator that does not rely on NO and, therefore, is an attractive agent for SCD-PH. Riociguat is currently approved for treatment of Group 1 PAH and Group 4 CTEPH. Weir et al. recently reported in a small case series tolerability of riociguat in four of six patients with sickle related CTEPH. Additionally, riociguat was associated with significant improvement in exercise capacity (mean 6MWD improvement of 56.8 m), functional class, NT-proBNP, and RVSP in 3 patients. Vaso-occlusive crisis developed in only one patient who was not on hydroxyurea (52). A phase 2, multi-multi-center, randomized, double-blind, placebo-controlled, study is now underway to evaluate the effectiveness and safety of riociguat in patients with sickle cell disease.

### Myeloproliferative Disorders

#### Prevalence

Myeloproliferative disorders (MPDs) are characterized by clonal expansion of the multipotent hematopoietic progenitor cell with overproduction of one of the blood elements (i.e., erythrocytes, leukocytes, or platelets). The prevalence of PH in patients with MPDs has been evaluated in multiple small studies. Most of these studies used Doppler echocardiography (PASP>35 mmHg) to diagnose PH, with an estimated prevalence of 13–48% of PH in MPDs (53–56). Interestingly, in a larger study of 103 patients with MPDs, the prevalence of PH was much lower with only 5 patients found to have PH (57). Among MPDs, patients with chronic myeloid leukemia, polycythemia vera, essential thrombocytosis, and myelofibrosis are particularly at higher risk of developing PH. The presence of PH is associated with poor prognosis in MPDs.

#### Pathogenesis

PH in MPDs has two main phenotypes: chronic thromboembolic (CTEPH) and proliferative pulmonary arteriopathy. Thrombotic events (both venous and arterial) and microcirculatory disturbances are common in polycythemia vera and essential thrombocytosis patients. In a large study of 1,213 patients with polycythemia vera, 41% developed thrombotic complications (58). This translates into higher risk of CTEPH. In a retrospective small study of 10 patients with MPDs and RHC-confirmed PH, six patients were diagnosed with CTEPH at the time of MPD diagnosis. CTEPH was strongly associated with elevated hematocrit (59). Presence of cardiovascular comorbidities, history of thrombosis or splenectomy and increased age further increase the risk of thrombosis in these patients (60–62).

High hematocrit in polycythemia vera is associated with hypercoagulability as demonstrated by the progressive increase in blood viscosity that parallels higher hematocrit values (63). The major impact of hematocrit on blood viscosity is in the venous (low-shear) circulation as it decreases blood flow and increases the risk of venous thrombosis. In the arterial circulation, under high-shear, platelets are displaced to the periphery by the large red cell mass and come in contact with endothelial cells, causing platelets activation (release of thromboxane A2, platelet-derived growth factor, vascular endothelial growth factor) and thrombosis (64, 65). The abnormalities of erythrocytes and platelets in MPDs also contribute to the aggregation of red blood cells and disturbed blood flow. Furthermore, chronically activated leukocytes (especially with JAK2 mutation) interact with platelets and endothelial cells and increase the risk of thrombosis (66–69). Splenectomy is used in the treatment of MPDs and can be associated with a hypercoagulable state as well (61). Notably, obstruction of pulmonary arteries in MPDs is not always related to thrombi but can be secondary to tumor emboli or circulating megakaryocytes with subsequent release of vasoactive substances that will further increase the tone in the pulmonary vascular bed (70, 71).

The second PH phenotype in MPDs is pulmonary arteriopathy which can result from different mechanisms. Portopulmonary hypertension may complicate chronic liver disease which is known to occur in MPDs (72). Medication-induced PH, fitting into Group 1 PH, is another potential cause of PH in these patients. Dasatinib, a tyrosine kinase inhibitor commonly used for treatment of chronic myeloid leukemia, has been associated with development of PH which seems to improve after discontinuation (73). Several TKIs other than dasatinib (e.g., bosutinib, ponatinib, lapatinib) have also been implicated in the development of PH. In a small study of 27 patients who received lapatinib, three patients were found to have PH by RHC with resolution of PH in all three after cessation of therapy (74).

Alkylating agents such as cyclophosphamide, melphalan, and busulfan (used for treatment of myelofibrosis) can cause PH with severely reduced diffusing capacity of the lung for carbon monoxide and abnormal findings on computed tomography of the chest (septal thickening, ground glass opacities, and lymph node enlargement) that are consistent with pulmonary veno-occlusive disease. Unlike dasatinib-induced PH, these patients have a poorer prognosis. Extramedullary hematopoiesis may complicate MPDs and result in infiltrative changes in the portal circulation with subsequent development of portal hypertension and portopulmonary hypertension. In cases of lung involvement, extramedullary hematopoiesis may lead to pulmonary vascular occlusion and PH. Proliferative changes in the pulmonary vessels may also occur due to increased circulating vascular endothelial growth factors and enhanced angiogenesis in MPDs (75). Finally, pre-capillary PH can be related to abnormal JAK2 signaling and depletion of NO. This is supported by improvement in PH and NO with JAK2 inhibitors.

#### Treatment

Since hematologic abnormalities contribute directly to the pulmonary vascular changes seen in MPDs, treatment of the underlying disease is a potential strategy. Indeed, allogeneic hematopoietic stem cell transplant and JAK inhibitors to treat MPDs have been shown to improve PH (76). For example, treatment with ruxolitinib (JAK1/JAK2 inhibitor) in 15 patients with myelofibrosis and PH resulted in improvement in right ventricular function and PASP by echocardiography in 66% of patients and was associated with significant reduction in NT-proBNP and increase in NO level (77). In cases of drug-induced PH, improvement in PH can be expected with discontinuation of the offending agent (especially in dasatinib-induced PH) although this alone may not be enough to reverse PH. When appropriate, CTEPH is treated with PTE or BPA. Riociguat, a guanylate cyclase stimulator, is associated with improved functional status and hemodynamics in patients with inoperable disease or suboptimal post-PTE hemodynamic outcomes (78, 79). External beam radiotherapy to the thorax has been used to treat extramedullary hematopoiesis in patients with MPDs and PH with improvement in pulmonary artery pressures (80). PAH-specific therapy has not been studied in MPD-associated PH.

## Pulmonary Hypertension in Sarcoidosis

### Prevalence

Sarcoidosis is characterized by granulomatous inflammation to an unknown trigger that primarily affects the lungs. The prevalence of sarcoidosis-associated PH (SAPH) is variable. This variation comes from inconsistency in methods used to diagnose SAPH. Autopsy studies have shown pulmonary vascular involvement in ~5% of patients with significant parenchymal lung disease (81). Similar prevalence has been found in studies that evaluated SAPH based on clinical evidence of right ventricular failure (i.e., cor pulmonale) (82). However, studies that used hemodynamic assessment using RHC to diagnose SAPH reported higher prevalence up to 23% (83, 84). The prevalence is substantially higher in patients with pulmonary symptoms and advanced lung disease due to sarcoidosis. In a study of 15 patients with advanced fibrotic sarcoidosis, Emirgil et al. found evidence of PH on RHC in 10 patients (67%) (85). Another study of 363 patients with advanced sarcoidosis (listed for lung transplantation) found evidence of PH on RHC in 74% of these patients (86). Racial differences may significantly impact the course of disease and risk of developing PH. For example, in a recent large prospective study of 512 patients, predominantly Caucasian, with sarcoidosis, SAPH was found in 2.9% of those with intermediate or high probability for PH (by echocardiography) (87). It is important to note that some patients with sarcoidosis may have normal pulmonary artery pressure at rest with evidence of exercise-induced PH (88).

### Pathogenesis

There are several mechanisms of PH in sarcoidosis. Each one of these may have a different response and outcome to a specific therapy. Pre-capillary PH may result from hypoxic pulmonary vasoconstriction and destruction of the distal vascular bed with advanced interstitial lung disease. This is supported by the finding that the majority of SAPH patients have advanced fibrotic lung disease (89–91). This usually leads to mild to moderate PH (mPAP <35 mmHg). Nevertheless, at least one third of sarcoidosis patients have no significant fibrosis which indicates that hypoxemia and fibrosis are not the only explanation for SAPH.

Granulomatous vasculopathy and angiitis with obliteration of arterioles or venules has been reported in autopsies of patients with pulmonary sarcoidosis (92–94). Occlusion of pulmonary vessels by granulomas causes PH and sometimes can be mistaken with thromboembolic disease. This pathology is associated with more severe PH than that related to lung fibrosis. Granulomatous vasculitis can also affect the post-capillary vessels causing focal stenosis and a clinical pattern consistent with pulmonary veno-occlusive disease (95, 96).

Moreover, sarcoidosis is associated with increased risk of venous thromboembolism, which carries associated risk of developing CTEPH in some patients (97, 98). Sarcoidosis can be associated with mediastinal and hilar lymphadenopathy that can cause extrinsic compression of pulmonary arteries (and venous stenosis) or fibrosing mediastinitis, resulting in SAPH.

Cardiac involvement occurs in 20–25% of patients with sarcoidosis (99). Thus, post-capillary PH can develop in sarcoidosis due systolic or diastolic dysfunction with left ventricular involvement. In a retrospective study of 130 sarcoidosis patients with persistent dyspnea, 29% of patients with SAPH had an elevated PAWP >15 mmHg (89). Because cardiac involvement can be subtle, screening for cardiac sarcoidosis using magnetic resonance imaging and 18 F-fluorodeoxyglucose positron emission tomography is recommended (100). Finally, post-capillary SAPH can be due to extrinsic compression of the pulmonary veins.

### Treatment

**Figure 3:**
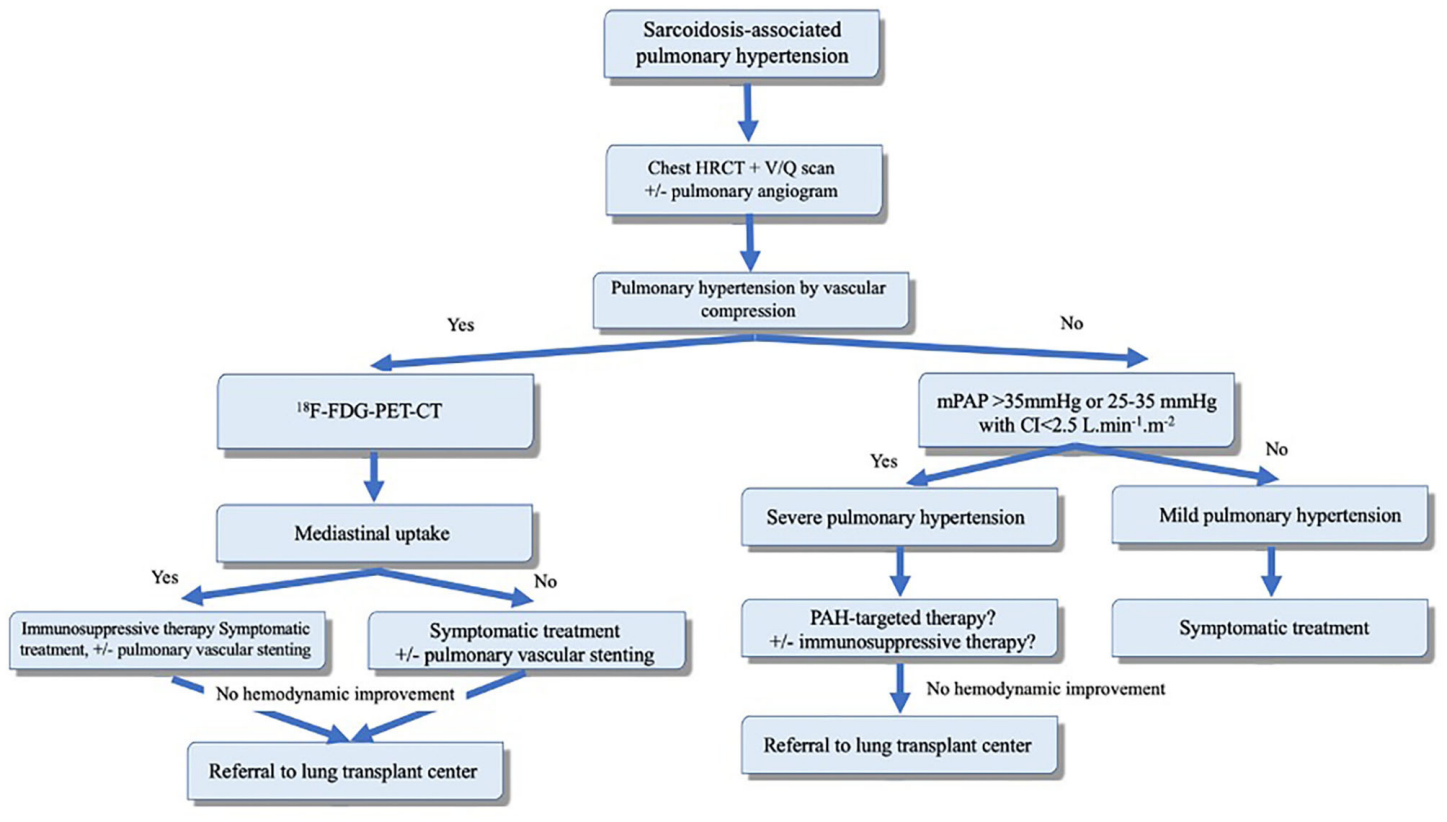
Proposed algorithm for evaluation and management of sarcoidosis-associated pulmonary hypertension (SAPH). Adapted from Boucly et al. (101). Reproduced with permission of the © ERS 2020.

As outlined earlier, SAPH can be related to multiple mechanisms (Figure 3). However, some patients may have a prominent mechanism. For example, in the presence of advanced parenchymal (fibrotic) lung disease, PH with mPAP of <35 mmHg is likely related to hypoxemia and destruction of the pulmonary vascular bed associated with fibrotic changes. Treatment in these patients should focus on treatment of parenchymal lung disease and correction of hypoxemia. PAH-specific therapy carries the risk of worsening ventilation-perfusion mismatch and, therefore, should be avoided.

PAH-specific therapy can be considered in carefully selected patients with moderate to severe SAPH that is “out-of-proportion” to the degree of parenchymal lung disease and hypoxia (i.e., mPAP >35 mmHg). Such degree of PH suggests a component of vasculopathy that may respond to pulmonary vasodilators. Vasoreactivity has been demonstrated in a small prospective observational study of 8 patients with moderate-to-severe SAPH with improvement in mPAP and PVR to inhaled NO (102). This highlights the potential role of pulmonary vasodilators in these patients. Such therapy has been evaluated in multiple studies and shown to improve the hemodynamic parameters with no significant change in functional status or 6-min walk distance (Table 3). Similarly, in a double-blind placebo-controlled trial, bosentan improved mPAP and PVR, but failed to demonstrate benefit in 6-min walk distance (110).

**Table 3:** Existing published studies evaluating treatment of sarcoid-associated pulmonary hypertension.

**Publication**	**Study design (number of subjects)**	**Treatment (# of patients)**	**Outcome**
Preston at al. (102)	Prospective observational (8)	Inh NO (5), inh NO with IV epo (1), CCB (2)	Short term 20% decreased PVR and mPAP; long term increased 6MWT
Culver et al. (103)	Retrospective chart review (7)	Bosentan (3), bosentan and IV epo (4)	Decreased mPAP at 6–18 mo in about 50% patients
Fisher et al. (104)	Retrospective case series (7)	IV epo (6), subcut trep (1)	Improved functional class
Milman et al. (105)	Retrospective chart review (12)	Sildenafil (12)	Decreased mPAP and PVR, increased CO, no change 6MWT
Barnett et al. (106)	Retrospective case series (22)	IV epo (1), bosentan (12), sildenafil (9)	Increased 6MWT and functional class, decreased mPAP and PVR
Baughman et al. (107)	Prospective open label 16 weeks (15)	Inh iloprost (15)	Decreased mPAP and PVR in 6 of 15 and increased 6MWT in 3 of 15 patients
Baughman et al. (89)	Retrospective chart review (5)	Bosentan (5)	Decreased mPAP in 3 of 5 patients at 4 mo
Judson et al. (108)	Prospective open label 12 weeks (25)	Ambrisentan (21)	No change 6MWT; 11 patients discontinued drug at 12 weeks
Dobarro et al. (109)	Retrospective chart review (8)	Sildenafil (9), bosentan (2); only 8 followed up with repeat RHC	Increased 6MWT and decreased NT-proBNP; non-statistically significant increase in CO/CI and decreased PVR
Baughman et al. (110)	Prospective placebo-controlled 16 weeks (35)	Bosentan (23), placebo (12)	Decreased mPAP and PVR; no change in 6MWT
Keir et al. (111)	Retrospective (33)	Sildenafil (29), bosentan (3)	Increased 6MWT, decreased NT-proBNP, improved TAPSE
Bonham et al. (112)	Retrospective case series (26)	Parenteral prostacyclin with epo (7) and trep (6), ERAs (12), PDE-5I (20), CCB (1)	Increased CI/CO, decreased PVR, improved functional class, decreased NT-proBNP
Ford et al. (113)	Prospective open label 24 weeks (12)	Tadalafil (12)	No change 6MWT at 24 weeks

*Inh, inhaled; NO, nitric oxide; IV, intravenous; epo, epoprostenol; CCB, calcium channel blocker; mPAP, mean pulmonary arterial pressure; PVR, pulmonary vascular resistance; 6MWT, 6 min walk test; CO, cardiac output; CI, cardiac index; mo, months; NT-proBNP, N terminal pro brain natriuretic peptide; TAPSE, tricuspid annular plane systolic excursion*.

Immunosuppressive therapy alone may result in hemodynamic improvement in patients with granulomatous vasculopathy or compressive lymphadenopathy (101). SAPH due to extrinsic compression can be treated with pulmonary artery or vein angioplasty and stenting (114). Overall, patients with SAPH have poor prognosis and should be evaluated early for lung transplantation.

## Pulmonary Hypertension in Chronic Kidney Disease and End Stage Renal Disease

The evaluation and management of PH in the context of chronic kidney disease (CKD), particularly end stage renal disease (ESRD), presents unique challenges to the clinician in terms of elucidating the main drivers of PH in this patient population. The clinical presentation of such patients is one in which PH is often multifactorial, hence the placement of this clinical entity into Group 5 of the PH classification schemata. As such, management relies in large part on attempts to correct and optimize multiple physiologic derangements often seen in CKD and ESRD.

### Definition, Epidemiology, and Scope of the Problem

CKD is defined as abnormal renal function present for at least 3 months and is staged in severity based on the degree of reduction of glomerular filtration rate and the degree of albuminuria. ESRD is defined as renal failure requiring regular interval, long-term dialysis, or renal transplant to maintain survival. The true prevalence of PH amongst patients with CKD and ESRD is difficult to discern, due to the lack of robust data. Most of what is known is drawn from relatively small, retrospective, and almost exclusively echocardiography based studies. Amongst patients with CKD and not yet on dialysis, the prevalence of PH was observed to be upwards of 30% or more. Prevalence was highest among those with most advanced CKD (CKD 5) (115, 116). When considering those patients on hemodialysis (HD), a meta-analysis of known studies by Tang and colleagues (116) showed the prevalence of PH (based largely on echocardiography) between 19 and 56%. There is less data available with regard to the prevalence of PH in the setting of peritoneal dialysis (PD), but it appears to be lower than that of HD (117).

**Figure 4:**
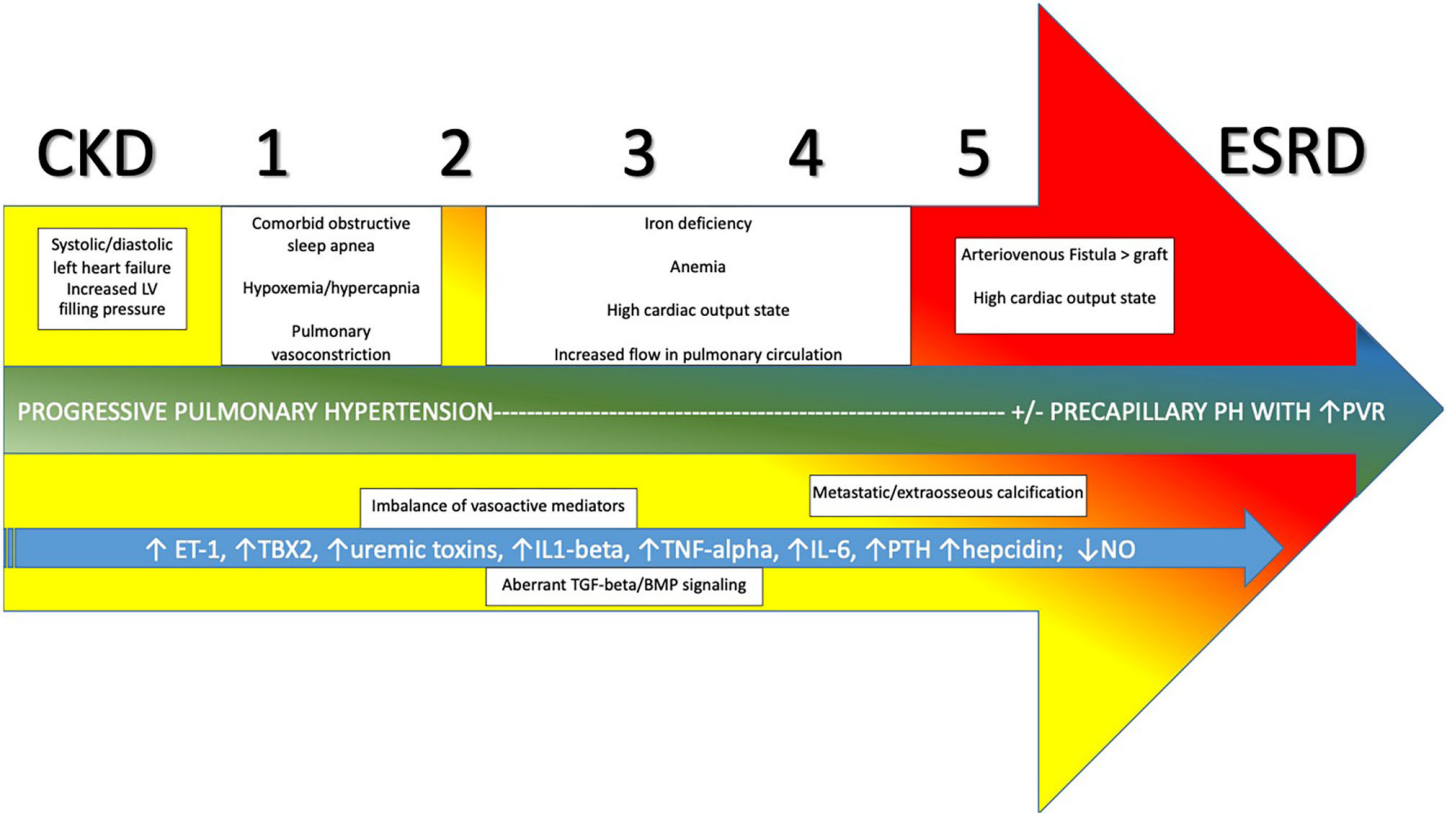
Evolution and development of pulmonary hypertension in CKD progressing through stages 1–5 to ESRD and relevant contributors. CKD, Chronic Kidney Disease; ESRD, End Stage Renal Disease; PVR, pulmonary vascular resistance; ET-1, endothelin-1; TBX2, thromboxane 2; IL1-beta, interleukin-1-beta; TNG-alpha, tumor necrosis factor-alpha; IL-6, interleukin-6; PTH, parathyroid hormone; NO, nitric oxide; TGF-beta, transforming growth factor-beta; BMP, bone morphogenetic protein.

O'Leary et al. (118) recently published a retrospective analysis of a large cohort of patients with varying degrees of CKD that underwent RHC. PH was present in 1,267/1,873 (68%) of patients, using the accepted definition of PH at that time of mean PAP ≥ 25 mmHg. Of these, 76% had post-capillary PH and 24% had pre-capillary disease, using elevated PAWP (≥ 15 mmHg) as the differentiating factor. The authors also noted that mortality was higher among patients with advanced stages of CKD, and further compounded by the presence of PH. The distribution of hemodynamic profiles seen underscores the significant role of Group 2 PH phenomenon related to elevated left ventricular filling pressures in driving PH in an overwhelming majority of patient with CKD. Comorbid left ventricular systolic and/or diastolic dysfunction, as well as a propensity to volume overload at times are the main drivers of this. However, many of those with CKD/ESRD with and without elevated PAWP have other factors driving PH as well. Among these are alterations in circulating inflammatory and vasoactive mediators, endothelial dysfunction, alterations in cardiopulmonary flow related to anemia and arteriovenous fistula (AVF), factors related to dialysis itself, and comorbid Group 3 PH processes (see Figure 4).

### Altered Inflammatory and Vasoactive Mediators and Related Endothelial Dysfunction

The presence of uremic toxins in patients with advanced CKD or ESRD can contribute to pulmonary vascular endothelial dysfunction. Specifically, mono-methyl L- Arginine, symmetric dimethyl arginine, and asymmetric dimethyl arginine are present in increased levels and inhibit NO synthase, impairing the intrinsic production and bioavailability of NO, the vasodilatory effects of which serve to maintain normal pulmonary vascular tone. There is also evidence that increased levels of uremic toxins are associated with increased inflammation, endothelial dysfunction, and oxidative stress (119). There is decreased availability of circulating NO in patients on HD with PH vs. those without PH. In addition, patients on HD with PH showed decreased ability to recover NO levels after HD, suggesting impairment in NO synthesis (120). As noted previously, ET-1 contributes to pulmonary vascular remodeling, is present in excess in patients with Group 1 PAH, and is also known to be elevated in patients with CKD and those on HD. Further, HD does not facilitate clearance of ET-1 and levels remain elevated after HD is performed (120). Angiopoietin 1 and 2 are additional mediators that modulate vascular smooth muscle growth via action on endothelial cells. There is evidence that both are important in the pathogenesis of CKD and PAH (121).

Cellular inflammation is a key driver of disease in both PH and CKD. In addition to the noted effects of uremic toxins, levels of circulating pro-inflammatory cytokines are increased in patients on HD with PH relative to those without, particularly IL-1beta, TNF-alpha, and IL-6. High-sensitivity c-reactive protein was also noted to be higher in the group with PH (122). The transforming growth factor beta (TGF-beta) and bone morphogenetic protein (BMP) signaling pathways modulate downstream gene expression that controls properties of vascular structure and tone. It has been demonstrated that this pathway is aberrant in CKD and PAH, leaning in a pro-inflammatory direction that favors vascular remodeling. Thus, there is a strong likelihood that the TGF-beta/BMP axis is relevant in patients with both PH and CKD/ESRD (123, 124).

### Alterations in Cardiopulmonary Flow

Intentional arteriovenous shunting caused by the surgical creation of AVF or placement of a synthetic arteriovenous graft (AVG) contributes to a high cardiac output state. This leads initially to increased loading on the left ventricle. When this phenomenon is coupled with the increased output state, a syndrome of high-output heart failure may manifest. With time, persistence of this high flow is thought to lead to increased shear forces on the pulmonary vascular endothelium that can cause pulmonary vascular remodeling and an intrinsic arteriopathy in some patients (125). The propensity to develop PH is higher in patients with AVF relative to AVG, as AVF can enlarge with time and develop progressively higher flows, which in turn increases flow through the pulmonary circulation to increasing degrees. It has been noted when flow through the fistula exceeds 2 L/min or more than 20% of cardiac output, the risk of pulmonary hypertension is higher. Furthermore, presence of the AVF on the upper arm presents increased likelihood of PH as compared to AVF of the lower arm (126).

Practices regarding the placement and maintenance of AVF for HD have come under a bit more scrutiny in recent years as a result of noted effects on circulatory and pulmonary vascular physiology. There has been deliberate advocacy on the part of the Centers for Medicare and Medicaid Services to utilize AVF over catheter-based HD in most patients, driven largely by legitimate concern for the risk of catheter related bloodstream infection and thrombosis (127). Since the inception of this initiative, it has been increasingly recognized that some patients with CKD/ESRD may have subclinical or mild PH before arterio-venous access for dialysis is placed. Some advocate for more systematic echocardiographic screening for PH prior to placement of AVF. If evidence of PH is present, then PD and/or expedited/prioritized renal transplant evaluation may be more appropriate (128). Further study is needed in this regard.

Anemia due to reduced renal production of erythropoietin, as well as iron deficiency and anemia of chronic disease is very common in patients with CKD and ESRD. This contributes to compensatory increase in cardiac output (much like that seen in SCD) and thus exposes the pulmonary circulation to higher than usual flow and the risks of vascular remodeling noted above. Iron deficiency in both PAH and CKD appears to be modulated largely by increased levels of hepcidin, which inhibits enteric iron transport and uptake (129, 130). Iron deficiency thus may ultimately affect pulmonary vascular regulation through downstream effects on hypoxia inducible factors. Risk for development of PH in the context of CKD/ESRD and anemia is most significant when hemoglobin is <10 g/dl (131).

### Factors Related to Dialysis

As noted, HD presents a higher likelihood of developing PH, due in part to the use of AVF or AVG in many patients. In addition, it appears that the type of dialysis membrane used may contribute to presence of PH as well. Thromboxane B2, a pro-proliferative, pro-thrombotic, and pro-inflammatory molecule, is known to be elevated in patients with PAH compared to controls. In addition, it has been shown that thromboxane B2 levels are higher in patients on HD and/or with PH as compared to those not on HD and/or with no evidence of PH (132). The reason for elevated thromboxane B2 in some patients on HD appears to be related to increased release from circulating monocytes when cellulose dialysis filter membranes are utilized as compared to polysulfone membranes (133, 134).

The likelihood of PH is also directly proportional to older age and number of years on HD (131). The reason for this is likely simply related to the duration of exposure of the pulmonary circulation to the aforementioned pathophysiologic effects of HD. An additional phenomenon that may contribute to pulmonary vascular changes and PH is the showering of micro-bubbles from the dialysis machine with each HD session. These micro-bubbles have the potential to obstruct small pulmonary capillaries, leading to local inflammatory changes and microthrombosis, setting the stage for progressive pre-capillary arteriopathy to progress as HD continues over time (135, 136).

### Comorbid Group 3 PH Processes

As noted previously, Group 3 PH is often driven by abnormal oxygenation and ventilation, commonly seen during sleep in patients with obstructive sleep apnea (OSA). There is a high prevalence of OSA amongst patients with CKD, independent of the need for HD (137, 138). The associated hypoxemia, and in some cases hypercapnia, contributes to pulmonary vasoconstriction and some degree of PH in many patients with CKD. Asymmetric dimethyl arginine is also noted to be elevated in patients with sleep disordered breathing, implicating impairment of NO production as at least one plausible molecular mechanism for the development of PH attributable to OSA (139). This pathway may be of particular relevance to patients with CKD. As such, screening for nocturnal hypoxemia and OSA with formal polysomnography is of paramount importance. Those found to have sleep disordered breathing should be titrated for and treated with nocturnal non-invasive positive airway pressure therapy.

In addition to OSA, it has been noted that secondary hyperparathyroidism seen in CKD/ESRD can lead to extraosseous calcium deposition in the interalveolar septae and pulmonary arterial system. Alveolar septal deposition can contribute to restrictive physiology on pulmonary function testing, as well as reduction in diffusing capacity (140). The overall clinical impact of these phenomenon with respect to the development of PH is not as clear compared to that of OSA. Additionally, it has been shown that parathyroid hormone levels do not differ between patients with and without PH in the context of CKD/ESRD (141).

### PH Diagnosis and Invasive Hemodynamic Evaluation in Patients With CKD/ESRD

As with all forms of pulmonary hypertension, the initial preferred tool for screening is transthoracic echocardiography. This is especially important in CKD/ESRD patients with unexplained dyspnea. Timing of echocardiography is most optimal soon after the completion of a session of dialysis, with the patient at or very near their established dry weight. The presence of estimated PASP ≥ 50 mmHg and or significant right ventricular dilatation or dysfunction should prompt invasive hemodynamic measurement by RHC. As with echocardiography, RHC should be performed in the relative immediate time period after a session of dialysis, such that the left and right sided circulation are as volume and pressure off-loaded as possible, ensuring that hemodynamic measurements are reflective largely of alterations in flow and/or PVR that may be present.

The PEPPER study (142) was an important endeavor that helped to understand the frequency of PH in patients with advanced CKD (stage 4 or 5) and unexplained dyspnea, as well as to understand the effect of HD on pulmonary hemodynamics. In the study, 31 CKD patients not on dialysis and 31 patients on HD with dyspnea underwent RHC. In the group on HD, 78% were found to have PH (13% pre-capillary PH, 65% post-capillary PH). Among those not on HD, 77% had PH (6% pre-capillary PH, 71% post-capillary PH). Further, in the group on HD, 25/31 underwent RHC pre and post dialysis. It was noted that mean PAP and PAWP were both significantly lower post-dialysis, underscoring the importance of timing of RHC relative to HD.

In patients that are on HD and have an AVF, it may be useful to study the effects of fistula occlusion during RHC, however there is scant literature to guide this practice. This must be done cautiously and for a relatively short time, as there is some risk of associated thrombosis of the AVF. Published studies looking at the hemodynamic effects of fistula compression are echocardiography based. Yigla et al. found that after compression of AVF for 1 min in four patients on HD with echocardiographic evidence of PH resulted in a 10% reduction in mean estimated PA systolic pressure and a 15% reduction in estimated cardiac output (reduction from supranormal values) (143).

### Treatment of PH in the Context of CKD and ESRD

Given that the majority of PH in the context of ESRD/HD is pulmonary venous hypertension, the mainstay of treatment is optimizing volume status with diuretics or dialysis, controlling systemic blood pressure, and medical therapies that optimize systolic and diastolic function of the left ventricle. The importance of correcting hypoxemia and sleep disordered breathing when relevant was previously discussed. If clinical history or risk factors suggest that CTEPH may be a possibility, then consideration must be given to screening ventilation-perfusion lung scan. If there is evidence of CTEPH, then additional imaging and hemodynamic workup is indicated to determine if the patient is a candidate for PTE or BPA. For patients in whom there is exceedingly high AVF flow and elevation in cardiac output driving PH, consideration should be given to revision of the AVF via banding or ligation (144, 145).

In general, trials of off label use of PAH-specific therapies in group 5 PH related to CKD/ESRD should be reserved for a very select few patients for whom all other contributing factors to PH have been optimized but have persistent and significant RHC-proven PVR elevation, particularly in the context of reduced cardiac output reflective of RV failure. The presence of clear Group 1 PAH comorbidities such as connective tissue disease, HIV, or portal hypertension, may also make a case for treatment with PAH therapies more compelling in the setting of a hemodynamic profile consistent with clear pre-capillary disease. As with all group 5 PH, there is no randomized, controlled data regarding the use of PAH-specific therapy in CKD/ESRD associated PH. Though not absolutely contraindicated in renal failure, PDE-5 inhibitors and riociguat should be used with caution in patients with advanced CKD/ESRD due to potential alterations in pharmacokinetics. As such, dose adjustments may be needed in this context. (146, 147).

Renal transplant surgery is generally regarded as not presenting prohibitive levels of operative risk to patients with mild to moderate degrees of PH and RV dysfunction, particularly considering the procedure is not usually associated with large intravascular volume and hemodynamic shifts as seen for example in liver transplantation. Patients with significant PH, and particularly severe right ventricular dysfunction, are considered with an abundance of caution for renal transplantation, especially if interventions to attempt to improve pulmonary hemodynamics have failed. There is no clear evidence based data available to systematically risk stratify patients with ESRD and PH for renal transplant surgery (148). Independent of immediate perioperative surgical risk however, it is noted that pre-transplant PH (based on echocardiographic studies) is associated with a significant increased risk of graft failure (149). Furthermore, post-transplant survival is decreased in patients with elevated estimated PASP on echocardiogram > 50 mmHg and in those with elevated PVR > 3 Wood units (150, 151). In general, approach to renal transplantation candidacy in the setting of PH should be done in an individualized, multidisciplinary fashion, especially given the lack of robust studies to reliably predict outcomes.

## Pulmonary Hypertension Associated With Less Common Comorbidities

### Pulmonary Langerhans Cell Histiocytosis

Pulmonary Langerhans cell histiocytosis (PLCH) is an uncommon cystic lung disease characterized by pathologic dendritic cell infiltration of the walls of the distal bronchiole. The involved dendritic cell subset, the Langerhans cell, localizes within the airway epithelium and processes encountered inhaled antigens, becoming activated when additional molecular signals indicate a state of danger (152). Activated dendritic cells migrate to lymphoid tissue to induce the adaptive immune response. PLCH occurs almost exclusively in smokers (153), and smokers with or without pulmonary diseases have significantly greater presence of Langerhans cells in the lung (154, 155). Cigarette smoking further induces production of molecules such as TNF-alpha, TGF-beta, GM-CSF, and osteopontin; these factors promote development, activation, and survival of Langerhans cells, though migration out of the airway tissue may be impaired (156–158). The bronchiolocentric nodules characteristic of PLCH contain a mixed population of Langerhans and other inflammatory cell types (159). Factors predisposing the small population of smokers developing PLCH remain incompletely understood.

Relative to other forms of chronic lung disease, PLCH is associated with higher prevalence of PH, and the vascular component of disease can be great (160, 161). PH has been found in 41–100% of PLCH patients, with prevalence particularly high (>90%) in those undergoing lung transplant evaluation (160, 162–164). Presence of PH has been consistently associated with increased mortality in PLCH (163, 165). While inverse correlation between pulmonary artery pressure elevation and spirometry has been noted in some studies (163, 166), others have not demonstrated this finding (160, 161). Examinations of tissue specimens have demonstrated typical vascular changes of PAH, including intimal fibrosis and medial hypertrophy of small and medium-sized pulmonary arteries (160). Observation of Langerhans cells with vessels is rare, and vascular involvement can also be noted in regions without parenchymal infiltration of Langerhans cells (160, 161). In contrast to most forms of PAH, capillary and venular pathology appears to be common in PLCH-PH (160, 165–168).

Management of PLCH, for those with or without associated PH, should most importantly center around smoking cessation. Stabilization and even substantial disease regression has been observed in those successful at quitting (169–172). While most case reports and series have focused on improvements in radiology or pulmonary function following smoking cessation, benefits on the pulmonary vascular component of disease should also be presumed. One case report of a patient with PLCH and associated PH has described PH resolution following smoking cessation (173). For patients with symptomatic PLCH refractory to smoking cessation, steroids, and chemotherapeutic agents have been used with mixed results (174–178). Use of PAH-specific therapy in those with PLCH-PH has been best described in a series by Le Pavec et al., where 14 patients were treated with an ERA, PDE-5I, and/or inhaled iloprost. Use of PAH-specific therapy was associated with improvements in mPAP and PVR on follow-up hemodynamic reassessments; a trend toward improved survival was also observed (167). With histologic observation of pulmonary venous involvement in PLCH-PH, the potential for development of pulmonary edema in response to PAH-specific therapy must be acknowledged, and cases consistent with this outcome have been documented after initiation of intravenous epoprostenol (160). Encouragingly, oxygenation of treated patients did not worsen in the series by Le Pavec (167). Given vascular complexity, lung transplant should be considered in those with associated PH and in others with severe PLCH. PLCH patients make up <1 percent of lung transplant recipients in the United States, with overall similar post-transplant outcomes to other patient groups (162, 164).

### Neurofibromatosis Type 1

Neurofibromatosis type 1 (NF1) is an autosomal dominant genetic disorder caused by mutation in NF1, the tumor suppressor gene encoding neurofibromin. Incidence is 1 in 3,000 births, with near complete penetrance; skin manifestations are usually prominent and early in onset. Major manifestations leading to diagnosis include café au lait macules, cutaneous and subcutaneous neurofibromas, axillary and inguinal freckling, and Lisch nodules of the iris (179). NF1-related parenchymal lung disease affects 10–20% of adults and is characterized by cysts, micronodules, and interstitial abnormalities (179–181). Arterial and venous vascular lesions, including arterio-venous malformations, arterial aneurysms, coarctation of the abdominal aorta, and renal artery stenosis, are important but under recognized sources of morbidity in NF1 (182, 183). Neurofibromin is expressed in endothelial and vascular smooth muscle cells, and it is thus suspected that absence of functional protein leads to dysregulation of growth and proliferation of intimal and medial layer cells, in systemic and pulmonary circulations (182, 184). Patients with NF1 also have increased malignancy risk, and cancer is the leading cause of death with life expectancy reduced by 10–15 years (185, 186).

Pulmonary hypertension in NF1 patients has been described only in case reports and series, thus prevalence of NF1-PH is difficult to estimate but thought to be rare. The largest series of PH in NF1 recently characterized 49 patients from the French PH National Referral Center. Despite NF1 affecting males and female equally, 80% of NF1-PH patients were female, and median age at diagnosis of PH was 62. No specific NF1 mutations were associated with PH, and others with NF1-PH were not observed in the families of most patients. Hemodynamics at diagnosis were consistent with severe pre-capillary PH (mean mPAP 45 with PVR 10.7 WU). The majority of patients had normal or mildly abnormal spirometry, with decreased diffusion capacity for carbon monoxide (median 30% predicted) and resting hypoxemia. Imaging was most frequently notable for diffuse lung cysts, ground glass opacities, and emphysema (187). Importantly, this series and another literature review have identified normal imaging or single radiologic abnormalities in nearly 30% of NF1-PH patients (184, 187). Lung tissue has been infrequently available; the few histologic studies have demonstrated arterial remodeling with muscularization of small arterioles as well as particular increase in intimal layer thickness (187, 188). Findings of thrombosis have not been noted, and presence of plexiform lesions has been variable (187, 189). Three patients undergoing transplant all had pulmonary venous wall thickening (187), and a patient with pulmonary capillary hemangiomatosis associated with NF1 has also been reported (190).

Prognosis of NF1-PH is poor, with transplant free survival of 87, 54, and 42% at 1, 3, and 5 years, respectively (187). Of patients in the large series by Jutant et al., 45/49 were started on PAH-specific therapy; ERAs, PDE-5Is, and prostacyclin derivatives were all used alone or in combination. Treated patients demonstrated improvement in cardiac index and PVR; however, oxygenation worsened slightly and clinical improvements were modest and not sustained (187). Lung transplantation has been successfully performed for NF1 patients (187, 188); however, concern for malignancy risk following transplant and immunosuppression can be a barrier (191). The tyrosine kinase inhibitor sorafenib, which is capable of suppressing kinases operating in pathways also shared by targets of neurofibromin, was used in a single patient with refractory NF1-PH. Hemodynamic and clinical improvements resulted after 3 months of use (192). Given neurofibromin's role in containment of cell proliferation, investigation of drugs impacting cell cycle regulation are particularly of interest in NF1-PH.

### Gaucher Disease

Gaucher disease (GD) is an autosomal recessive lysosomal storage disease with an incidence of 1 in 50,000 births. GD results from inadequate function of the lysosomal enzyme glucocerebrosidase, leading to accumulation of its substrate in macrophages, described as Gaucher cells. Accumulation of macrophages is particularly prominent in the bone marrow, liver, and spleen, with resulting hepatosplenomegaly, thrombocytopenia, anemia, osteopenia, and painful bone crises. GD is classified into three subtypes (GD 1, 2, and 3), with GD type 1, the non-neuronopathic form of GD, being the most common. Pulmonary involvement can be seen in all GD types, though those with GD1 are at most risk for developing pulmonary vascular complications of the disease (193). Enzyme replacement therapy for GD was first approved by the United States Food and Drug Administration in 1991 and has provided significant improvements in hematologic counts, spleen and liver volumes, and other clinical parameters for GD type 1 (194, 195). Importantly, use of enzyme replacement therapy has dramatically decreased need for splenectomy, previously performed for GD due to lack of other management options (195–197).

The prevalence of PH in adult GD type 1 patients, as estimated by echocardiography, ranges from 7 to 30% (198, 199). Mild PASP elevation (35–50 mmHg) is noted in the majority, with 0.76% documented to have PASP >50 (198). Treatment with enzyme replacement therapy is associated with lower prevalence of PH (7.4% in treated patients vs. 30% in untreated patients) (198). Though enzyme replacement therapy has been questioned to be causative of PH (200, 201), most current evidence suggests protection, and multiple examples of PH-related clinical improvements after initiation exist (198). In contrast, splenectomy has strong association with development of GD-PH. Severe PH complicating GD occurs predominantly in females who have undergone splenectomy as part of GD management (198, 199). The largest published series of GD-PH patients included 14 adult patients from a single referral center. Females made up 71% of the population, with a mean age of 36 at PH diagnosis, and all patients had previously undergone splenectomy at mean age 12. Other pertinent findings from this series includes lack of correlation between development of PH and GD severity by other metrics. Heteroallelic mutations (with one copy of the most common mutation N370S) were more prevalent than N370S homozygotes (199). Of interest, this report and others provide examples of patients also meeting criteria for hepatopulmonary syndrome; in these cases PH was either coexisting with hepatopulmonary syndrome or was unmasked after initiation of enzyme replacement therapy and resulting resolution of hepatopulmonary syndrome (199, 202). When tissue has been available, observed arterial changes have resembled those of PAH, including medial hypertrophy and intimal fibrosis, as well as plexiform lesions (203–205). In addition, intravascular Gaucher cells have been observed, though are not uniformly present (205–207).

If not previously initiated, most GD-PH patients will start on enzyme replacement therapy, with case reports of this alone leading to significant improvement (198, 199). ERAs, PDE-5Is, and prostanoids have all been used in individual patients with GD-PH, generally with positive outcomes (198, 199, 208). Given shared risk factor (splenectomy) with CTEPH, use of anticoagulation should be discussed particularly in splenectomized patients, though data about its role is lacking. Coumadin has been used in several patients in combination with enzyme replacement therapy and PAH-specific therapy (198). In cases of refractory GD-PH, imatinib has been used successfully in a single patient (209). Finally, though other comorbidities can pose a challenge to candidacy, lung transplant has been used as a treatment modality for a few cases of severe GD-PH (203, 205).

## Discussion

Group 5 pulmonary hypertension remains a varied and challenging group of clinical entities. Understanding of the pathogenetic factors underlying many of the clinical conditions in the group has advanced considerably, however the relative rarity and/or variable clinical presentation of many of these disease processes has made it challenging to identify unifying treatment principles that can be applied broadly. Greater understanding may be gained by enhanced prospective registries of the more common conditions associated with PH, particularly sarcoidosis, sickle cell disease, myeloproliferative disorders, and chronic kidney disease/ESRD. More robust randomized trials of PAH therapies will be needed to understand if any of the ever-increasing number of PAH treatments may be of benefit in any of these populations.

## Author Contributions

All authors listed have made a substantial, direct and intellectual contribution to the work, and approved it for publication.

## Conflict of Interest

The authors declare that the research was conducted in the absence of any commercial or financial relationships that could be construed as a potential conflict of interest.
